# The quadruplex fluorescent quantitative PCR method for the simultaneous detection of respiratory diseases in quail: *Pasteurella multocida*, *Avibacterium paragallinarum*, *Mycoplasma gallisepticum*, and *Mycoplasma synoviae*

**DOI:** 10.3389/fmicb.2025.1605356

**Published:** 2025-06-30

**Authors:** Haojie Wang, Lihong Xue, Longxi Wang, Yixuan Liu, Jianxing Chen, Yue Sun, Tongqing An, Changwen Li, Hongyan Chen, Changqing Yu, Changyou Xia, He Zhang

**Affiliations:** ^1^State Key Laboratory for Animal Disease Control and Prevention, Harbin Veterinary Research Institute, Chinese Academy of Agricultural Sciences, Harbin, China; ^2^School of Advanced Agricultural Sciences, Yibin Vocational and Technical College, Yibin, China

**Keywords:** *Pasteurella multocida*, *Avibacterium paragallinarum*, *Mycoplasma gallisepticum*, *Mycoplasma synoviae*, quadruplex fluorescent

## Abstract

**Background:**

The quail farming industry constitutes an important component of China’s agricultural sector. However, it is frequently threatened by various bacterial and mycoplasmal infections, particularly respiratory diseases caused by *Pasteurella multocida*, *Avibacterium paragallinarum*, *Mycoplasma gallisepticum*, and *Mycoplasma synoviae*. These pathogens commonly result in co-infections or secondary infections, and their clinical presentations are often indistinguishable due to the similarity of symptoms.

**Methods:**

Four sets of primers and probes were designed based on the GenBank-registered gene sequences: the *kmt1* gene of *P. multocida*, the *recN* gene of *A. paragallinarum*, the *mgc2* gene of *M. gallisepticum*, and the *vlhA* gene of *M. synoviae*. Reaction conditions were optimized accordingly. A recombinant plasmid standard was constructed for the generation of standard curves. The sensitivity, specificity, reproducibility, and accuracy of the assay were systematically evaluated.

**Results:**

The constructed standard curves demonstrated strong linearity (*R*^2^ = 1.000, 0.998, 1.000, and 1.000), with high amplification efficiencies (107.09, 91.23, 112.10, and 125.51%, respectively). The detection limit for each recombinant plasmid standard was as low as 10 copies. No cross-reactivity was observed with non-target pathogens, including avian pox virus, *Escherichia coli*, *Salmonella* spp., Newcastle disease virus, infectious bronchitis virus, infectious laryngotracheitis virus, and *Staphylococcus aureus*. The assay exhibited excellent reproducibility, with inter- and intra-assay coefficient of variation (CV) values ranging from 0.11 to 1.41%. Among 126 clinical samples, *P. multocida* was detected in 6 samples, *A. paragallinarum* in 3, *M. gallisepticum* in 6, and *M. synoviae* in 4. These results were consistent with those obtained using previously established methods.

**Discussion:**

A highly sensitive, specific, rapid, and efficient quadruplex fluorescence quantitative PCR assay was successfully developed for the simultaneous detection and identification of *Pasteurella multocida*, *Avibacterium paragallinarum*, *Mycoplasma gallisepticum*, and *Mycoplasma synoviae*.

## 1 Introduction

The poultry industry is a vital component of China’s agricultural economy, with quail farming occupying a significant position both nationally and globally. According to industry statistics from 2025, the population of egg-laying quail in China is estimated at approximately 500 million, with an annual production exceeding 1.5 million tons of quail eggs, accounting for over 60% of global output (Gui et al., 2024). Major quail farming regions are concentrated in provinces such as Henan, Shandong, Anhui, Jiangsu, and Hebei, where large-scale production clusters have been established (Gui et al., 2024; Wang et al., 2012; Rigobelo et al., 2013). However, the frequent occurrence of bacterial and mycoplasmal infections poses a serious threat to the sustainable development of the quail industry in China. Respiratory diseases alone account for a mortality rate ranging from 10 to 40% in affected flocks (Rigobelo et al., 2013). Due to the common co-occurrence of bacterial and mycoplasmal pathogens, accurate diagnosis and effective control remain challenging. The primary etiological agents associated with quail respiratory diseases include *Pasteurella multocida* (*P. multocida*, Pm), *Avibacterium paragallinarum* (*A. paragallinarum,* Apg), *Mycoplasma gallisepticum* (*M. gallisepticum,* MG), and *Mycoplasma synoviae* (*M. synoviae,* MS), all of which are also recognized as major pathogens in chickens, ducks, and other poultry species (Balouria et al., 2019; Williams et al., 2000; Fair et al., 1999; He et al., 2024).

*P. multocida* is a gram-negative, non-spore-forming, non-motile short rod bacterium capable of growth under both aerobic and facultatively anaerobic conditions (Allen et al., 2024). It is a primary pathogen responsible for acute respiratory infections in poultry, exhibiting particularly high pathogenicity in chickens, ducks, and quail (Allen et al., 2024; Poussard et al., 2025; Bathobakae et al., 2025). Based on capsular antigen composition, *P. multocida* is classified into several serotypes (A, B, D, E, F), with serotypes A and F most commonly associated with fowl cholera (Poussard et al., 2025; Bathobakae et al., 2025). In quail, infection typically manifests as acute respiratory disease characterized by high fever, nasal discharge, drooling, and tachypnea, which may rapidly progress to septic shock and death in severe cases.

*A. paragallinarum,* a gram-negative bacterium belonging to the genus Avibacterium, grows under both aerobic and anaerobic conditions and requires V factor (NAD) supplementation for *in vitro* culture (Boguslavsky et al., 2000). This pathogen is transmitted primarily via direct contact or aerosol routes and causes respiratory symptoms in poultry, including coughing, nasal discharge, conjunctivitis, and reduced egg production. In some instances, affected birds may exhibit depression and anorexia (Huo et al., 2023).

*M. gallisepticum*, known as a major chicken pathogen, is a gram-negative bacterium with stringent growth requirements (Mugunthan et al., 2023). It has a broad host range encompassing mammals, reptiles, and birds, and is transmitted through both vertical and horizontal routes (Zhang et al., 2025). In quail, infection commonly presents with nasal discharge, coughing, air sac thickening, and purulent exudates.

*M. synoviae,* also referred to as synovial fluid mycoplasma, is another significant pathogen causing respiratory disease in poultry (Feberwee et al., 2022). Transmission occurs both vertically and horizontally, with horizontal spread via airborne particles, direct contact, and contamination of feed or water sources (Feberwee et al., 2022). Infected quail exhibit wheezing, nasal discharge, depression, swelling of the footpads, and enlargement of the hock and toe joints. Additionally, *M. synoviae* infection can negatively impact egg production, egg quality, hatchability, and feed conversion efficiency (Feberwee et al., 2022).

Co-infection with *P. multocida*, *A. paragallinarum*, *M. gallisepticum*, and *M. synoviae* is common in poultry, and diagnosis based solely on clinical symptoms is challenging (Wu et al., 2024; Chaidez-Ibarra et al., 2022; Wu et al., 2025). Conventional laboratory diagnostic methods, such as bacterial isolation and animal inoculation tests, are complex, time-consuming, costly, and exhibit relatively low sensitivity, limiting their utility for rapid clinical diagnosis. Consequently, there is an urgent need for rapid and accurate diagnostic methods capable of detecting mixed infections of multiple pathogens (Abate and Fentie, 2023; Yadav et al., 2022). Fluorescence quantitative PCR (qPCR) technology, characterized by high sensitivity, specificity, and throughput, has become a valuable tool for pathogen detection (Wang et al., 2024). However, existing assays for these four pathogens are generally designed for single or dual pathogen detection, lacking multiplex methods capable of simultaneous identification, which reduces detection efficiency and increases costs.

In this study, we developed a high-throughput, highly sensitive, specific, and accurate quadruplex qPCR assay for the simultaneous detection of *P. multocida*, *A. paragallinarum*, *M. gallisepticum*, and *M. synoviae*. This method provides a robust technological platform for veterinary clinical diagnosis, poultry health management, and the sustainable development of the poultry industry.

## 2 Materials and methods

### 2.1 Nucleic acids and clinical samples

Nucleic acids from avian pox virus, *Escherichia coli*, *Salmonella* spp., Newcastle disease virus, infectious bronchitis virus, infectious laryngotracheitis virus, *Staphylococcus aureus*, *Pasteurella multocida*, *Avibacterium paragallinarum*, *Mycoplasma gallisepticum*, and *Mycoplasma synoviae* were stored in our laboratory. Between July 2024 and March 2025, a total of 126 clinical samples—including nasal swabs, cloacal swabs, feces, and tissue samples (heart, lungs, and trachea)—were collected from quail farms located in Beijing and Jiangxi provinces. Samples were obtained from both clinically healthy quails and individuals exhibiting symptoms such as fever, dyspnea, and depression. Importantly, no additional harm or invasive procedures were performed on the animals as part of this study. Given the nature of the research, the Institutional Review Board of the Harbin Veterinary Research Institute determined that ethical approval was not required.

### 2.2 Reagents and instruments

The 2 × Taq Probe qPCR-Multiplex kit (Cat. No. B630005-0005) was purchased from Sangon Biotech (Shanghai) Co., Ltd. Plasmid mini prep kits (Cat. No. M1261-00) were obtained from Omega Bio-tek. The bacterial genomic DNA extraction kit (Cat. No. ATC-DNA) and viral RNA/DNA extraction kit (Cat. No. ATC-D/RNA) were purchased from Jinrui Hongjie (Xiamen) Biotechnology Co., Ltd.

### 2.3 Processing of clinical samples and nucleic acid extraction

Tissues including the heart, lungs, and trachea were collected and placed into sterile centrifuge tubes containing an appropriate volume of phosphate-buffered saline (PBS). The tissues were homogenized using an automated tissue grinder, followed by three freeze–thaw cycles. Cloacal and nasal swabs, as well as environmental samples, were thoroughly mixed and subjected to three freeze–thaw cycles. Fecal samples were suspended in sterile centrifuge tubes containing 1 mL of PBS and vigorously shaken for 1 min. Genomic DNA and RNA were extracted from all samples following the protocols provided by the manufacturers of the respective commercial kits.

### 2.4 Primer and probe design

Based on the GenBank reference sequences of the *P. multocida kmt1* gene (AF067175), *A. paragallinarum recN* gene (DQ899748.1), *M. gallisepticum mgc2* gene (NC_018406.1), and *M. synoviae vlhA* gene (CP011096.1), four pairs of specific primers and probes were designed (Table 1). All primers and probes were synthesized by Harbin Qingke Biotechnology Co., Ltd.

**Table 1:** Primer and probe information for the four pathogens.

Pathogens	Gene	Sequence of primer and probe (5′-3′)	Production
*P. multocida*	*kmt1*	F: TGACAGCTTTGTGATCTGGATTG R: GTCACTCTACTGGCGCGTTAAA Probe: FAM-TTTGCCACGCGAATT-MGB	66 bp
*A. paragallinarum*	*recN*	F: TCACAAACCTTTCGCAATCG R: TGGATTGTGCGGTAGAGCAA Probe: NED-TTAAATACCCTCAGTGAAAAC-MGB	85 bp
*M. gallisepticum*	*mgc2*	F: TACGAACATTCACCCACACTTGT R: CCAGCACCTGCACCCACTA Probe: VIC-ATGAAGGTGAAACTAATTC-MGB	110 bp
*M. synoviae*	*vlhA*	F: AACAGATGGTGCTTTACCAAACC R: AACAGATGGTGCTTTACCAAACC Probe: Cy5-AACCAAAGCTAGAGATAAA-MGB	92 bp

### 2.5 Preparation of the quadruplex recombinant plasmid standard

The target gene fragments from the four pathogens were synthesized by Harbin Qingke Biotechnology Co., Ltd. and sequentially cloned into the pMD-18 T vector to construct a quadruplex recombinant plasmid. Following PCR amplification and sequencing, the recombinant plasmid was confirmed to be accurate and was designated as pMD-kmt1-recN-mgc2-vlhA. The plasmid standard was then prepared in large quantities, and its concentration was measured using a UV spectrophotometer. The quadruplex recombinant plasmid standard was stored at −20°C until further use. The plasmid copy number was calculated using the following formula: Plasmid copy number (copies/μL) = (6.02 × 10^23^) × [Plasmid concentration (ng/μL) × 10^9^] / (DNA length × 660) (Wang et al., 2024).

### 2.6 Optimization of reaction conditions

The quadruplex recombinant plasmid standard pMD-kmt1-recN-mgc2-vlhA was used as the template to optimize the reaction conditions. A matrix experiment was conducted employing four specific primer and probe sets. The total reaction volume was 20 μL, and annealing temperatures of 59°C, 60°C, 61°C, and 62°C were tested. The working concentration of all primers and probes was initially set at 10 μM. The volumes of primers and probes for Pm-F, Pm-R, Pm-P, HPG-F, HPG-R, HPG-P, MG-F, MG-R, MG-P, MS-F, MS-R, and MS-P were systematically varied between 0.1 μL and 0.5 μL to identify the optimal concentrations for the quadruplex fluorescence quantitative PCR assay.

### 2.7 Construction of standard curve and sensitivity validation

The quadruplex recombinant plasmid standard was serially diluted 10-fold from 1 × 10^9^ copies/μL to 1 × 10^0^ copies/μL and used as the template. Amplification was performed under the optimized reaction conditions, and a standard curve was generated to evaluate the sensitivity of the assay.

### 2.8 Specificity test

Nucleic acids from avian pox virus, *Escherichia coli*, *Salmonella* spp., Newcastle disease virus, avian infectious bronchitis virus, infectious laryngotracheitis virus, *Staphylococcus aureus*, *P. multocida*, *A. paragallinarum*, *M. gallisepticum*, and *M. synoviae* were used as templates. The quadruplex recombinant plasmid standard pMD-kmt1-recN-mgc2-vlhA served as the positive control, while ultrapure water was used as the negative control. The optimized quadruplex fluorescence quantitative PCR assay was applied to evaluate the specificity of the method.

### 2.9 Reproducibility test

The quadruplex recombinant plasmid standard was serially diluted 10-fold, and three concentrations (1 × 10^7^, 1 × 10^5^, and 1 × 10^3^ copies/μL) were selected for amplification using the optimized quadruplex fluorescence quantitative PCR assay. Inter- and intra-assay reproducibility were assessed to determine the consistency and reliability of the method.

### 2.10 Clinical samples detection

The quadruplex fluorescence quantitative PCR assay developed in this study, together with a previously established fluorescence quantitative PCR method for detecting the four pathogens, was applied to test 126 clinical samples from quail. The concordance between the two methods was analyzed to evaluate the practical applicability of the developed assay.

## 3 Results

### 3.1 Optimal reaction conditions for the quadruplex fluorescence quantitative PCR assay

After optimization of annealing temperature, primer and probe concentrations, and cycle number, the optimal reaction conditions for the quadruplex fluorescence quantitative PCR assay were established as follows: the total reaction volume was 20 μL, containing 10.0 μL of 2 × Taq Probe qPCR-Multiplex. The final concentrations of primers and probes for *P. multocida*, *A. paragallinarum*, *M. gallisepticum*, and *M. synoviae* were 0.05 μM and 0.05 μM, 0.125 μM and 0.05 μM, 0.075 μM and 0.10 μM, and 0.125 μM and 0.075 μM, respectively. A plasmid template concentration of 1 × 10^7^ copies/μL was used, and ddH₂O was added to complete the volume. Regarding the cycle number, amplification beyond 40 cycles resulted in high background fluorescence, whereas fewer than 40 cycles led to insufficient amplification. The thermal cycling protocol was as follows: initial denaturation at 95°C for 3 min, followed by 40 cycles of denaturation at 95°C for 10 s and annealing/extension at 60.0°C for 30 s, with fluorescence data collected during the extension phase.

### 3.2 Standard curve construction and sensitivity evaluation

**Figure 1:**
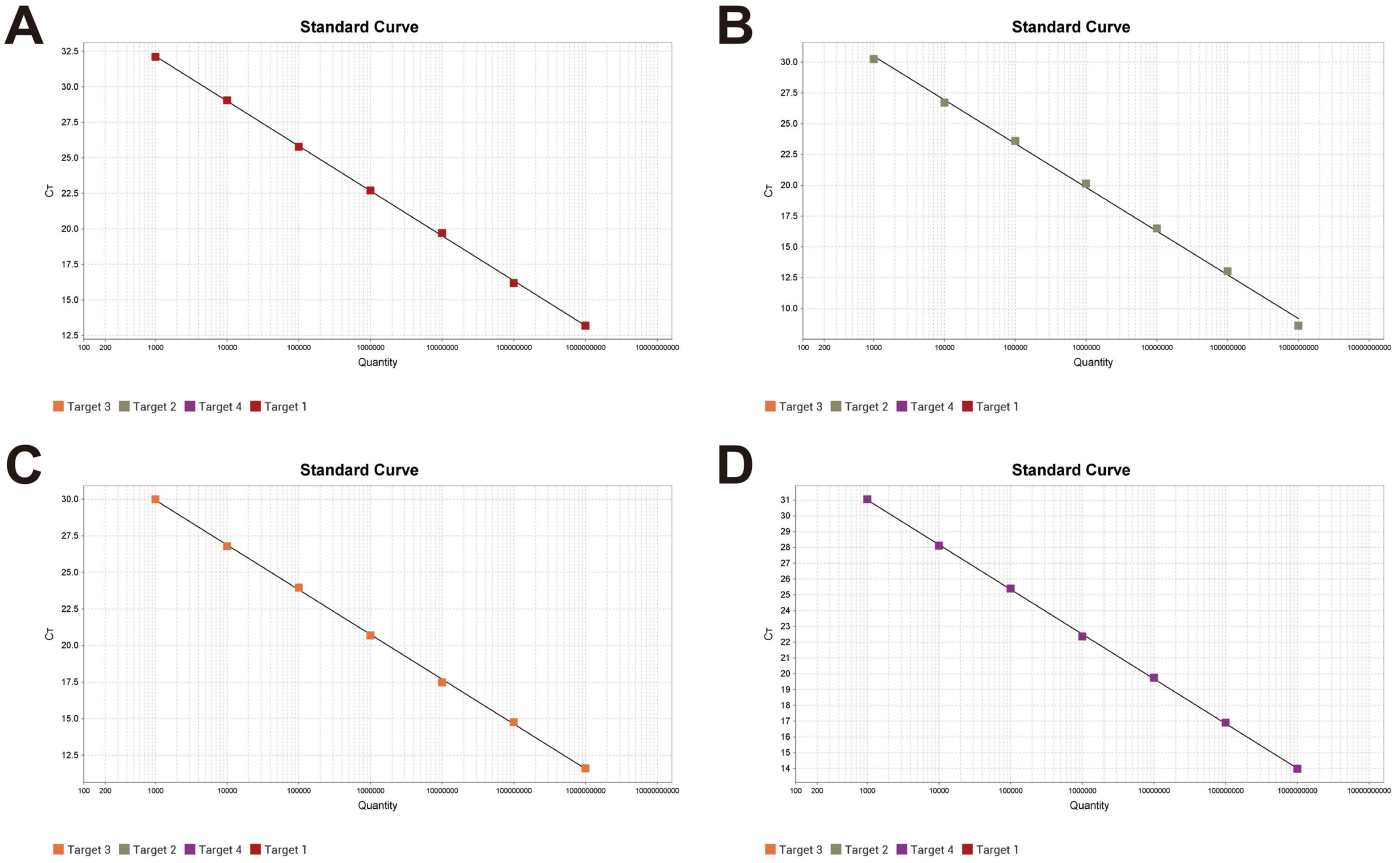
Standard curves of the quadruplex fluorescence quantitative PCR method X-axis: Plasmid copy number; Y-axis: Ct value. **(A)** The linear equation for Pm standard curve: *Y* = −3.163lg(X) + 41.654, *R*^2^ = 1, EFF% = 107.088. **(B)** The linear equation for Apg standard curve: *Y* = −3.552lg(X) + 41.145, *R*^2^ = 0.998, EFF% = 91.226. **(C)** The linear equation for MG standard curve: *Y* = −3.062lg(X) + 39.131, *R*^2^ = 1, EFF% = 112.095. **(D)** The linear equation for MS standard curve: *Y* = −2.832lg(X) + 39.501, *R*^2^ = 1, EFF% = 125.512.

The quadruplex recombinant plasmid standard was serially diluted 10-fold from 1 × 10^9^ to 1 × 10^0^ copies/μL and used as the template for amplification following the optimized quadruplex fluorescence quantitative PCR protocol. A standard curve was constructed over the concentration range of 1 × 10^9^ to 1 × 10^3^ copies/μL, as shown in Figure 1. The correlation coefficients (R^2^) for all four pathogens exceeded 0.990, indicating a strong linear relationship between template concentration and Ct values.

**Figure 2:**
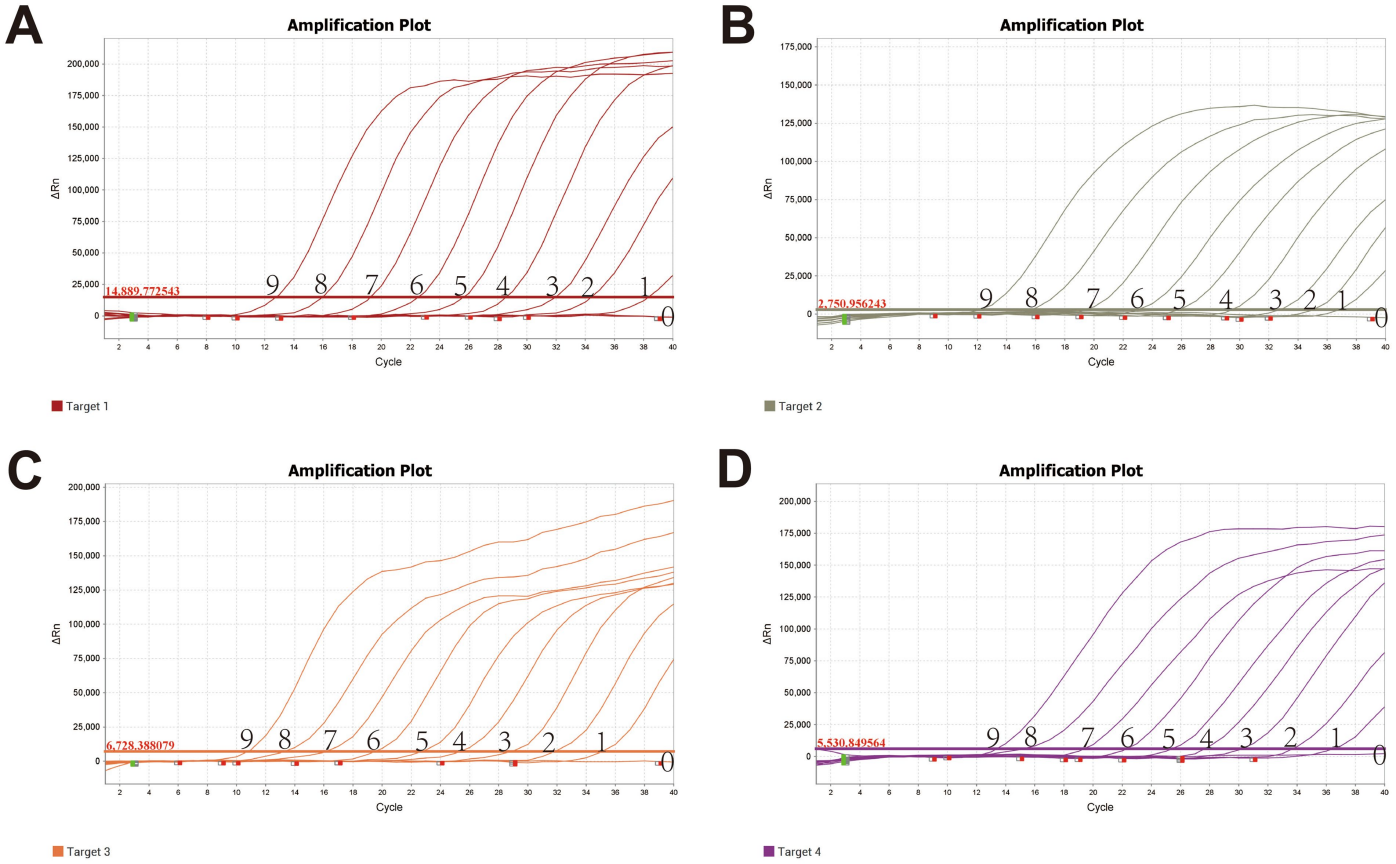
Sensitivity validation of the quadruplex fluorescence quantitative PCR Method X-axis: Ct value; Y-axis: Fluorescence signal intensity. **(A)** Pm; **(B)** Apg; **(C)** MG; **(D)** MS; Lanes 0–9 represent quadruplex plasmid concentrations ranging from 10^9^ copies/μL to 10^0^ copy/μL.

The limit of detection for the optimized assay, determined using plasmid standards at different dilutions, was 10 copies (Figure 2), demonstrating high sensitivity. Positive controls for *P. multocida*, *A. paragallinarum*, *M. gallisepticum*, and *M. synoviae* (detected via FAM, NED, VIC, and Cy5 channels, respectively) exhibited typical sigmoid amplification curves. Negative controls (FAM, NED, VIC, and Cy5) showed no amplification, with Ct values ≥40 or undetermined. The assay was considered valid when these criteria were met. Samples with Ct values <36 and typical amplification curves were classified as positive. Samples with Ct values between 36 and <40 were considered suspicious and were retested in duplicate. Samples with Ct values ≥40 or undetermined and no typical amplification curve were classified as negative.

### 3.3 Specificity results

**Figure 3:**
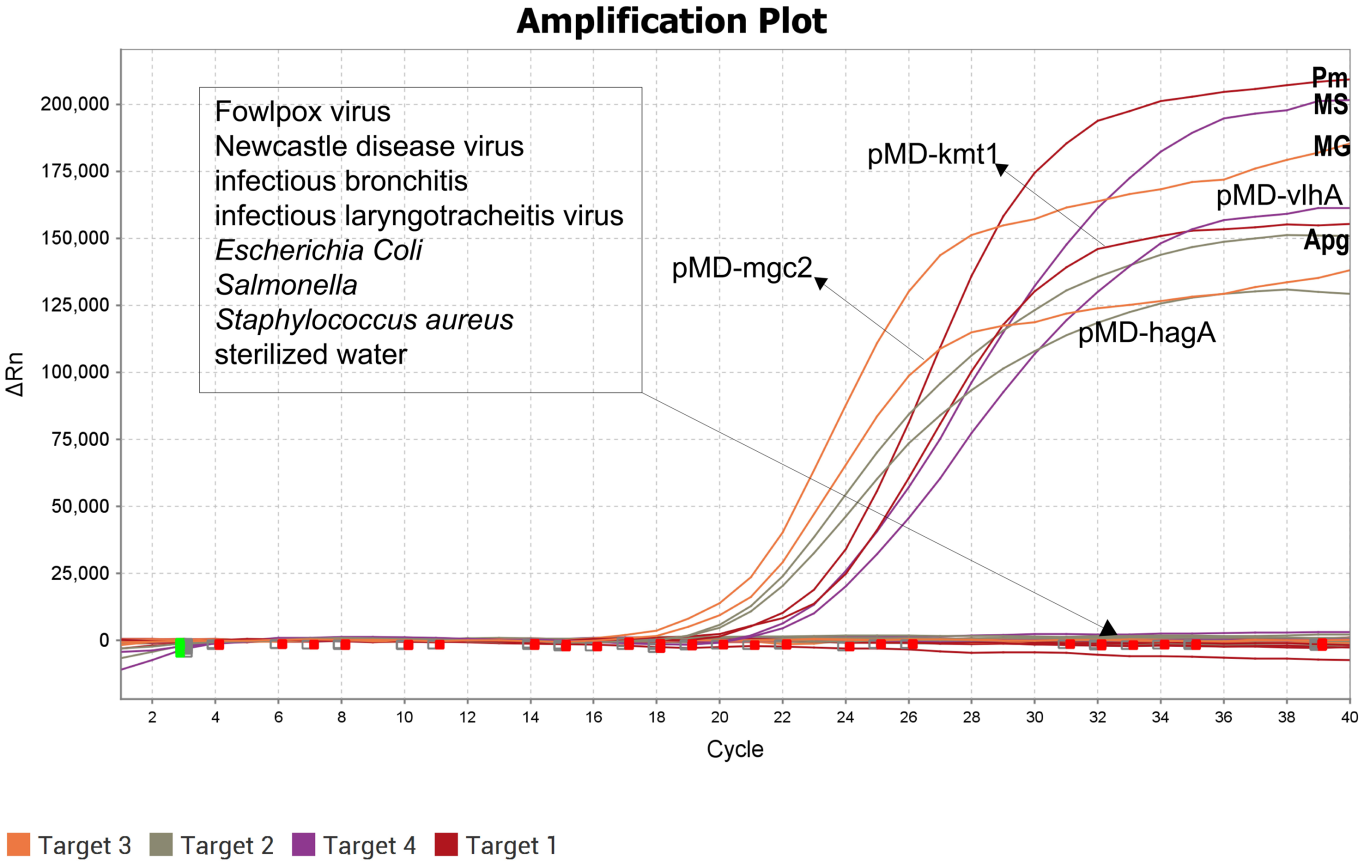
Specificity validation of the quadruple fluorescence quantitative PCR method.

The optimized quadruplex fluorescence quantitative PCR assay was used to test nucleic acids from Fowlpox virus, *Escherichia coli*, *Salmonella* spp., Newcastle disease virus, Infectious bronchitis virus, Infectious laryngotracheitis virus, *Staphylococcus aureus*, *P. multocida*, *A. paragallinarum*, *M. gallisepticum*, and *M. synoviae*. The results showed that amplification curves were produced exclusively for the target pathogens, with no cross-reactivity observed among non-target organisms. These findings demonstrate that the established assay possesses high specificity (Figure 3).

### 3.4 Repeatability results

The optimized quadruplex fluorescence quantitative PCR assay was evaluated for intra- and inter-assay repeatability using plasmid standard samples at final concentrations of 1 × 10^7^, 1 × 10^5^, and 1 × 10^3^ copies/μL. The coefficient of variation (CV) of Ct values across these concentrations ranged from 0.11 to 1.41% (Table 2), demonstrating excellent repeatability and consistency of the assay.

**Table 2:** Intra- and inter-batch validation results of the quadruplex fluorescence quantitative PCR method.

Standard plasmid	Concentration of template (copies/μL)	Intra-coefficient of variation		Inter-coefficient of variation	
		X ± SD	CV (%)	X ± SD	CV (%)
pMD-kmt1	10^7^	19.523 ± 0.021	0.11	19.482 ± 0.014	0.07
	10^5^	25.869 ± 0.061	0.24	25.771 ± 0.102	0.40
	10^3^	32.115 ± 0.130	0.40	32.185 ± 0.098	0.30
pMD-recN	10^7^	16.081 ± 0.112	0.70	16.341 ± 0.085	0.52
	10^5^	23.285 ± 0.108	0.46	23.181 ± 0.327	1.41
	10^3^	30.459 ± 0.221	0.73	30.201 ± 0.109	0.36
pMD-mgc2	10^7^	17.667 ± 0.167	0.94	17.543 ± 0.087	0.50
	10^5^	23.411 ± 0.128	0.55	23.341 ± 0.091	0.39
	10^3^	29.485 ± 0.152	0.52	29.846 ± 0.163	0.55
pMD-vlhA	10^7^	19.567 ± 0.066	0.34	19.317 ± 0.083	0.43
	10^5^	25.221 ± 0.144	0.57	25.614 ± 0.197	0.77
	10^3^	31.105 ± 0.105	0.34	31.251 ± 0.127	0.41

### 3.5 Clinical sample detection results

**Figure 4:**
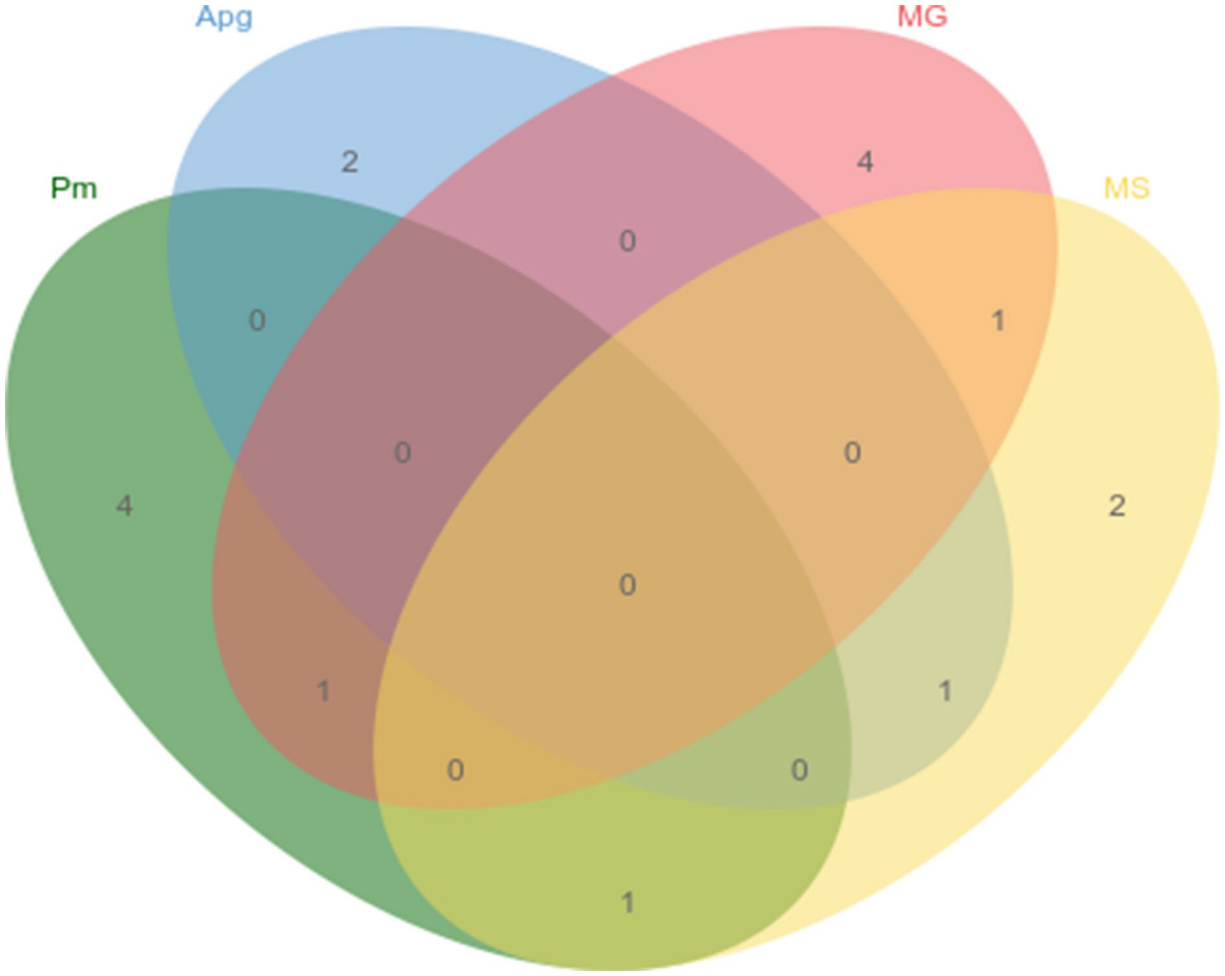
Single and mixed infection status of positive samples.

The newly established quadruplex fluorescence quantitative PCR assay, alongside previously reported PCR methods for *P. multocida*, *A. paragallinarum*, *M. gallisepticum*, and *M. synoviae*, was applied to test 126 clinical samples from quail. The detection rates were 4.8% for *P. multocida*, 2.4% for *A. paragallinarum*, 4.8% for *M. gallisepticum*, and 4.0% for *M. synoviae*, with detailed co-infection patterns illustrated in Figure 4. Furthermore, the results obtained with the quadruplex assay were 100% concordant with those from the established methods, confirming the high accuracy and reliability of the developed assay.

## 4 Discussion

Quail is an economically important poultry species in China, playing a vital role in the agricultural industry (He et al., 2023). Its eggs and meat are highly valued for their nutritional benefits and health-promoting properties (Mo et al., 2013). However, respiratory diseases caused by *P. multocida*, *A. paragallinarum*, *M. gallisepticum*, and *M. synoviae* severely threaten quail health and productivity, leading to significant economic losses. These pathogens induce a range of respiratory conditions, chronic airsacculitis, and occasionally mortality. Moreover, co-infections and secondary infections with these agents exacerbate disease severity and complicate clinical management (Zhang et al., 2015). Due to the overlap in clinical symptoms caused by these pathogens, accurate and rapid diagnosis remains challenging, impeding effective prevention and control. Hence, there is an urgent need for a sensitive, specific, and high-throughput diagnostic tool capable of timely and simultaneous detection of these pathogens.

In this study, we addressed this need by targeting conserved and specific gene sequences of the four pathogens. Multiple primer and probe sets were designed and rigorously screened to develop a quadruplex fluorescence quantitative PCR assay for simultaneous detection. The selection of target genes was fundamental to the assay’s performance, influencing sensitivity, specificity, and overall accuracy. For *P. multocida*, commonly used targets include *16S rRNA*, *kmt1*, capsular genes, and virulence factors (Davies, 2004; Sun et al., 2021; Wang et al., 2023). Given the diversity of *P. multocida* serotypes infecting poultry, especially types A, D, and F, genes with broad serotype coverage were prioritized (Allen et al., 2005). Although the *16S rRNA* gene is frequently used, primers targeting this region showed cross-reactivity in preliminary tests (data not shown). The *kmt1* gene, known for its specificity and stability, has been widely used in PCR, LAMP, and RPA assays, and was selected as the optimal target (Poussard et al., 2025; Alemu et al., 2023; Hao et al., 2023).

*A. paragallinarum* consists of serotypes A, B, and C, each with potentially distinct genetic markers (Tan et al., 2021). Previous studies identified *hagA*, *lysS*, and *recN* as candidate targets (Gallardo et al., 2020; Krylova et al., 2023; Wen et al., 2016). The *recN* gene, a conserved housekeeping gene across all serotypes, was chosen to ensure comprehensive detection (Wen et al., 2016). Importantly, despite homologous sequences in related bacteria, sequence divergence in *recN* ensures assay specificity.

For *M. gallisepticum*, the genome encodes over 700 proteins, including virulence factors such as adhesion and membrane proteins (Mugunthan et al., 2023). Among adhesion genes, *mgc2*, *GapA*, and *PvpA* have been utilized diagnostically (Boguslavsky et al., 2000; Bao et al., 2015; Ruger et al., 2022; Lysnyansky et al., 2005). The *mgc2* gene was selected due to its established reliability in molecular detection.

*Mycoplasma synoviae* carries over 650 protein-coding genes, with common diagnostic targets including *vlhA*, *NOX*, and *Eno* (Si et al., 2023). The *vlhA* gene stands out for its specificity and prevalence in phylogenetic analyses, and has gained prominence in recent diagnostic developments (Slavec et al., 2011). Accordingly, *vlhA* was selected. The quadruplex qPCR assay demonstrated a detection limit of 10 copies per reaction for recombinant plasmid standards from all four pathogens. This sensitivity compares favorably with or surpasses previously reported qPCR assays, which have detection limits ranging from 14 to 7,000 copies depending on the pathogen and method (Sun et al., 2021; Krylova et al., 2023; Grodio et al., 2008; Huang et al., 2015).

Key performance metrics—sensitivity, specificity, repeatability, and accuracy—were systematically evaluated. Optimization expanded pathogen detection capacity without compromising sensitivity. Specificity testing against nucleic acids from other common quail pathogens confirmed no cross-reactivity, underscoring high assay specificity. Repeatability assessments showed coefficients of variation below 2% for intra- and inter-assay measurements, confirming robustness. Clinical validation using 126 quail samples yielded detection rates consistent with previous reports and demonstrated 100% concordance with established assays. Notably, the assay reliably identified co-infections, which is critical for comprehensive clinical diagnosis and management.

Nevertheless, limitations exist. Epidemiological data on these respiratory pathogens in quail remain scarce, both domestically and globally. The relatively small clinical sample size in this study may limit the generalizability of prevalence estimates. Future work should involve larger, geographically diverse sample collections to better characterize pathogen distribution and to validate the assay’s utility across different clinical settings. Such efforts will facilitate enhanced surveillance, early diagnosis, and effective control measures to mitigate the impact of respiratory diseases in the quail industry.

## 5 Conclusion

This study successfully identified and selected conserved, pathogen-specific gene sequences of *P. multocida*, *A. paragallinarum*, *M. gallisepticum*, and *M. synoviae*. Based on these targets, specific primers and probes were designed, followed by comprehensive optimization of reaction conditions and system parameters. The result is a sensitive, specific, and accurate quadruplex fluorescence quantitative PCR assay capable of simultaneously detecting all four pathogens. This method exhibits excellent sensitivity, specificity, repeatability, and stability, providing a powerful tool for the early diagnosis and prevention of respiratory diseases in quail. Furthermore, by generating valuable epidemiological data, this detection platform will support precise disease monitoring and contribute to the sustainable development and effective management of the quail farming industry.

## Data Availability

The original contributions presented in the study are included in the article/supplementary material, further inquiries can be directed to the corresponding authors.
